# Proposal for the reclassification of obligately purine-fermenting bacteria *Clostridium acidurici* (Barker 1938) and *Clostridium purinilyticum* (Dürre *et al.* 1981) as *Gottschalkia acidurici* gen. nov. comb. nov. and *Gottschalkia* *purinilytica* comb. nov. and of *Eubacterium angustum* (Beuscher and Andreesen 1985) as *Andreesenia angusta* gen. nov. comb. nov. in the family *Gottschalkiaceae* fam. nov.

**DOI:** 10.1099/ijsem.0.002008

**Published:** 2017-08-15

**Authors:** Anja Poehlein, Natalya Yutin, Rolf Daniel, Michael Y. Galperin

**Affiliations:** ^1^​ Department of Genomic and Applied Microbiology and Göttingen Genomics Laboratory, Institute of Microbiology and Genetics, Georg-August-University, Göttingen, Germany; ^2^​ National Center for Biotechnology Information, National Library of Medicine, National Institutes of Health, Bethesda, MD 20894, USA

**Keywords:** *Clostridium*, taxonomy, sporulation, purine degradation, 16S rRNA, uric acid

## Abstract

Several strictly anaerobic bacteria that are Gram-stain-positive have the ability to use uric acid as the sole source of carbon and energy. The phylogeny of three such species, *Clostridium acidurici*, *Clostridium purinilyticum*, and *Eubacterium angustum*, members of the *Clostridium* cluster XII that ferment purines, but not most amino acids or carbohydrates, has been re-examined, taking advantage of their recently sequenced genomes. Phylogenetic analyses, based on 16S rRNA gene sequences, protein sequences of RpoB and GyrB, and on a concatenated alignment of 50 ribosomal proteins, revealed tight clustering of *C. acidurici* and *C. purinilyticum. Eubacterium angustum* showed consistent association with *C. acidurici* and *C. purinilyticum* , but differed from these two in terms of the genome size, G+C content of its chromosomal DNA and its inability to form spores. We propose reassigning *C. acidurici* and *C. purinilyticum* to the novel genus *Gottschalkia* as *Gottschalkia acidurici* gen. nov. comb. nov. (the type species of the genus) and *Gottschalkia purinilytica* comb. nov., respectively. *Eubacterium angustum* is proposed to be reclassified as *Andreesenia angusta* gen. nov. comb. nov. Furthermore, based on the phylogenetic data and similar metabolic properties, we propose assigning genera *Gottschalkia* and *Andreesenia* to the novel family *Gottschalkiaceae.* Metagenomic sequencing data indicate the widespread distibution of organisms falling within the radiation of the proposed family *Gottschalkiaceae* in terrestrial and aquatic habitats from upstate New York to Antarctica, most likely due to their ability to metabolize avian-produced uric acid.

For historical reasons, the genus *Clostridium* includes a large number of diverse bacteria whose only common features are obligately anaerobic growth, a Gram-positive type cell wall, the absence of sulfate reduction and the ability to form endospores [1–3]. In 1994, based on the studies of clostridial 16S rRNA gene sequences, Collins and colleagues divided it into 19 clusters that roughly represented family-level taxa; each cluster included several proposed genera [4]. Over the past 20 years, many former *Clostridium* spp. have been reassigned to new genera, some have been moved to novel families, orders and even to the novel classes, *Erysipelotrichia* and *Negativicutes* [1, 5]. An important step towards streamlining clostridial classification has been made in the latest edition of Bergey’s Manual of Systematic Bacteriology [1, 6], which reclassified a large number of *Clostridium* spp. based on phylogenetic criteria, along the lines of the work of Collins *et al*. [4].

In 2016, Lawson and Rainey [7] proposed limiting the genus *Clostridium* to the members of *Clostridium sensu stricto* (*Clostridium* cluster I [4]), which includes approximately 70 species that are sufficiently close to the type species *Clostridium butyricum*. Adoption of this proposal means that species of the genus *Clostridium* that do not belong to cluster I need to be reclassified. Here, we propose such a reclassification for three species of bacteria with validly published names, *Clostridium acidurici*, *Clostridium purinilyticum* and *Eubacterium angustum*, members of the *Clostridium* cluster XII [4, 8]. Based on the phylogenetic analyses presented here and in a previous work [9], we propose re-assigning these three organisms to two novel genera, *Gottschalkia* and *Andreesenia*, within the novel family *Gottschalkiaceae*.

In their original description of *Clostridium* cluster XII, Collins *et al*. [4] identified two loosely connected branches. One of them included a tight cluster of *C. acidurici* and *C. purinilyticum*, which shared approximately 94 % similarity with respect to 16S rRNA gene sequences and were put into the same genus. A subsequent paper from the same authors added *E. angustum* to the same genus [8]. The other branch included *Clostridium hastiforme, Clostridium* sp. strain BN11, and ‘*Clostridium filamentosum*’. The first two were later reclassified as *Tissierella praeacuta* and *Tissierella creatinini*, respectively [8, 10]. ‘*Clostridium filamentosum’* has not been validly named but is available under this name in some culture collections (e.g., ATCC 25785 = JCM 6585). Based on its 16S rRNA gene sequence, it probably belongs to the genus *Anaerosalibacter* and is listed as *Anaerosalibacter* sp. in the DSMZ catalog (https://www.dsmz.de/catalogues/details/culture/DSM-6645.html). Because of the ambiguous phylogeny of *Tissierella*-related organisms, in the 2009 edition of Bergey’s Manual of Systematic Bacteriology these organisms, along with the members of *Clostridium* cluster XIII, were assigned to *Clostridiales* Family XI *Incertae Sedis* [6]. More recently, members of cluster XIII have been assigned to the family *Peptoniphilaceae* [11], whereas the genera *Tissierella and Soehngenia* (and potentially also *Sporanaerobacter* and *Tepidimicrobium*) have been proposed to form the novel family *Tissierellaceae* in the order *Tissierellales* [12]. These changes still left three members of the original *Clostridium* cluster XII without a correct assignment: *C. acidurici*, *C. purinilyticum* and *E. angustum* [4, 8], and these are the subjects of the present study.

Phylogenetic analyses, based upon the 16S rRNA gene sequences of *C. acidurici*, *C. purinilyticum* and *E. angustum* and their neighbours from clusters I, XI, XII, and XIII were performed using the neighbour-joining (Fig. 1) and maximum likelihood methods (Fig. S1, available in the online Supplementary Material). The 16S rRNA gene sequences of the type strains were obtained either from GenBank or from the NCBI RefSeq Targeted Loci project [13] (see the online Supplementary Material for details). Sequences were aligned with ClustalW [14], as implemented in the mega7 software suite [15], and the neighbour-joining and maximum likelihood trees were reconstructed using mega7.

**Fig. 1.**
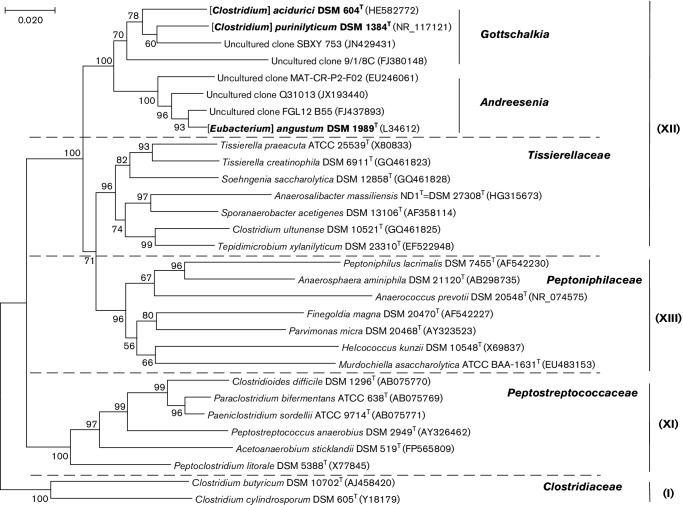
16S rRNA gene-based phylogenetic tree of *Clostridium acidurici* and related organisms and metagenomic samples. The names of the characterized members of the proposed genera *Gottschalkia* and *Andreesenia* are shown in bold in square brackets. The sequences from type strains (indicated with ^T^) were used and listed under their DSM accession numbers; where available. GenBank accession numbers are listed in parentheses. Roman numerals on the right indicate the clostridial cluster assignments of Collins *et al.* [4]. *Clostridioides difficile*, *Acetoanaerobium sticklandii* and *Peptoclostridium litorale* are the recently assigned names of formerly misclassified *Clostridium* spp. [45, 46]. The tree was inferred using the neighborhood-joining method, based on the Tamura-Nei model [47] as implemented in mega7 [15]. The evolutionary distances were computed using the Jukes-Cantor method and are in the units of the number of base substitutions per site. The tree was rooted using sequences from *C. butyricum* and *C. cylindrosporum*, which are members of *Clostridium sensu stricto* (cluster I).

The 16S rRNA gene-based phylogenetic trees (Figs 1 and S1) showed that *C. acidurici*, *C. purinilyticum* and *E. angustum* form a distinct cluster, separate from other species of cluster XII (members of *Tissierellaceae*), as well as from representatives of clusters I, XI, and XIII (members of *Clostridiaceae, Peptostreptococcaceae* and *Peptoniphilaceae,* respectively). As noted previously, *C. acidurici* and *C. purinilyticum* are particularly closely related [4, 16–18]. *E. angustum* forms a separate branch in the same cluster, as it did in the trees presented in several earlier reports [2, 8, 19–21].

To further evaluate the phylogenetic relationships of *C. acidurici*, *C. purinilyticum* and *E. angustum*, we have analyzed protein trees reconstructed from ribosomal proteins (Fig. 2) and from sequences of the DNA-directed RNA polymerase beta subunit (RpoB) and DNA gyrase subunit B (GyrB) of various members of clostridial clusters I, XI, XII, and XII, using sequences from selected organisms with completely or partially sequenced genomes, where available (Table S1). The ribosomal proteins-based phylogenetic tree was reconstructed from a concatenated alignment of 50 widespread ribosomal proteins, as described earlier [9, 22], (see online Supplementary Material for details). On this tree, *C. acidurici*, *C. purinilyticum* and *E. angustum* again formed a tight cluster with well-supported branches (Fig. 2). Clustering of these organisms was also seen in the phylogenetic trees for RpoB and GyrB subunits (Fig. S2a, b). The assignment of *C. acidurici* and *C. purinilyticum* to a single genus satisfies both rRNA similarity-based [23] and protein overlap-based [24] criteria. Based on these data, we formally propose reassigning *C. acidurici* and *C. purinilyticum* to the novel genus *Gottschalkia*.

**Fig. 2.**
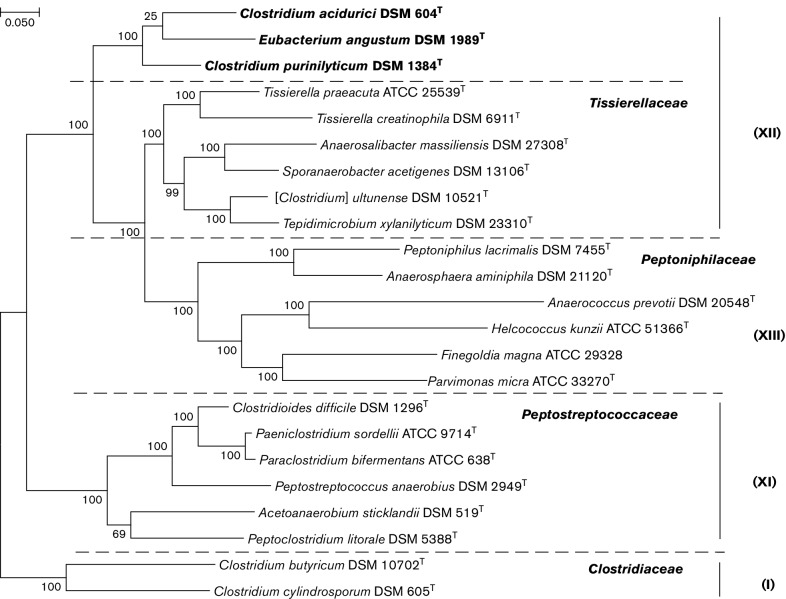
Ribosomal protein-based phylogenetic tree of *Clostridium acidurici* and related species. Members of the proposed genera *Gottschalkia* and *Andreesenia* are shown in bold. Roman numerals on the right indicate the clostridial cluster assignments of Collins *et al.* [4]. The tree was reconstructed essentially as described previously [9, 22]. Fifty sets of ribosomal proteins (L1–L7, L9–L11, L13–L24, L27–L29, L31–L36 and S2–S20) were extracted from the respective genomic entries (listed in Table S1) and aligned using muscle [48]; gapped columns (with more than 30 % of gaps) and columns with low information content were removed from the alignments. Individual ribosomal protein alignments were concatenated, giving a total of 6238 positions, and a maximum-likelihood tree was reconstructed using the PhyML program [49], the latest version of which (http://www.atgc-montpellier.fr/phyml-sms/) includes automatic selection of the best-fit substitution model for a given alignment and calculation of branch support values using aBayes algorithm [50]. The tree was rooted using the sequences from *C. butyricum* and *C. cylindrosporum*.

While unification of *C. acidurici* and *C. purinilyticum* has already been proposed by Collins *et al*. [4] and appears quite straightforward, *E. angustum* differs from them in having a much higher DNA G+C content, a smaller genome size, and an inability to form spores (Table 1). While the ability to sporulate is not necessarily a reliable taxonomic character [8, 25], as it can be easily lost through a deletion of a single core sporulation gene [12, 26], the smaller genome size of *E. angustum* compared to its relatives (Table 1) indicates substantial loss of genes in its particular lineage. However, *E. angustum* still encodes certain sporulation proteins, although far fewer than *C. acidurici* and *C. purinilyticum* (Table S2). *E. angustum* has been reported to be non-motile, but formed flagella [27] and its genome carries more than 30 flagellar genes [28]. Based on the differences listed above, and the lower level of similarity in its 16S rRNA gene sequence (91 %) than that recommended for a single genus [23], *E. angustum* does not fit into the genus *Gottschalkia.* Further, the percentages of conserved proteins between *E. angustum* and *C. acidurici* and *C. purinilyticum*, calculated as described by Qin and colleagues [24] (49.2 and 47.7 %, respectively) were lower than the suggested genus boundary of 50 %. Accordingly, we propose placing *E. angustum* in a separate genus, *Andreesenia*.

**Table 1.** Characteristics of *Clostridium acidiurici*, *Clostridium purinilyticum* and *Eubacterium angustum*, members of the proposed novel genera *Gottschalkia and Andreesenia* 1*, Clostridium acidurici* 9a^T^=DSM 604^T^ [16, 29]; 2, *Clostridium purinilyticum* WA-1^T^*=*DSM 1384^T^ [16, 43]; 3, *Eubacterium angustum* MK-1^T^=DSM 1989^T^ [27, 28]; 4, *Tissierella praeacuta* ATCC 25539^T^ or *Tissierellacreatinophila* KRE 4^T^=DSM 69113^T^ [8, 12, 19, 51]; 5, *Soehngeniasaccharolytica* BOR-Y^T^=DSM 12858^T^=ATCC BAA-502^T^ [20]; 6, *Anaerosalibacterbizertensis* C5BEL^T^=DSM 23801^T^ or *Anaerosalibacter* sp. ND1=DSM 27308 [21, 52]; 7, *Clostridium ultunense* DSM 10521^T^ [53, 54]; 8, *Clostridium cylindrosporum* HC1^T^=DSM 605^T^ [16, 29, 55–57]. ± , weak or variable reaction; nd, no available data.

**Property**		**Organisms**							
		**1**	**2**	**3**	**4**	**5**	**6**	**7**	**8**
Genome size (kb)		3108	3397	2405	3116	nd	3198	3217	2720
Proteins encoded		2774	3135	2397	2957	nd	3054	2863	1879
DNA G+C (mol%)		29.9	28.8	43.7	30.1	43	29.7	32.8	27.9
Cell width (µm)		0.5–0.7	1.1–1.6	1.0–1.5	0.6–0.9	0.5–0.7	0.5–1.0	0.5–0.7	0.8
Cell length (µm)		2.5–4.0	2.7–9.6	3.0–6.5	2–8	2–11	3–20	0.5–7.0	3.3
Gram staining		+	+	+	±	+	+	–	±
Flagella		+	+	+	±	+	+	+	+
Spore formation		+	+	–	±	+	+	+	+
Optimal temperature for growth (**°C)**		31–37	36	37	37	30–37	40	37	40–45
Optimal pH for growth		7.6–8.1	7.3–7.8	8.0–8.2	7.5	7.0	7.5	7.0	7.0–8.0
Hydrolysis of									
Gelatin		–	–	–	±	–	+	–	–
Starch		–	–	–	–	+	–	nd	–
Utilization of purines									
Adenine		–	+	–	–	nd	nd	nd	–
Adenosine		–	+	–*	nd	nd	nd	nd	–
Guanine		+	+	+	nd	nd	nd	nd	+
2-Hydroxypurine		+	+	–*	nd	nd	nd	nd	–
Hypoxanthine		+	+	–†	nd	nd	nd	nd	+
Purine		+	+	–*	nd	nd	nd	nd	–
Uric acid		+	+	+	–	nd	nd	nd	+
Xanthine		+	+	+	–	nd	nd	nd	+
Xanthosine		–	+	–*	nd	nd	nd	nd	–
Utilization of sugars									
l-Arabinose		–	–	–*	–	+	–	–	–
Cellobiose		–	–	–*	–	+	–	–	–
d-Fructose		–	–	–*	–	+	–	–	–
d-Galactose		–	–	–*	–	+	–	–	–
d-Glucose		–	–	–*	–	+	+	+	–
Lactose		–	–	–*	–	+	+	–	–
Maltose		–	–	–*	–	+	–	–	–
d-Mannitol		–	–	–*	–	+	+	–	–
d-Mannose		–	–	–*	–	+	–	–	–
d-Ribose		–	–	–*	–	+	–	–	–
d-Sorbitol		–	–	–*	–	+	nd	–	–
Sucrose		–	–	–*	–	+	–	–	–
d-Xylose		–	–	–*	–	+	nd	–	–
Enzymes									
Catalase		–	–	–	–	–	–	–	–
Lecithinase		–	–	–	–	nd	nd	nd	–
Lipase		–	–	–	–	nd	nd	nd	–
Urease		–	–	–	–	–	–	nd	–
Production of									
Acetate		+	+	+	+	+	+	+	+
Butyrate		–	–	–	+	nd	+	–	–
Formate		+	+	+	–	+	–	+	+
CO_2_		+	+	+	+	+	nd	+	+
NH_3_		+	+	+	+	±	nd	nd	+
H_2_		–	–	–	nd	+	–	+	–
H_2_S		nd	–	–	+	+	–	nd	–
Reduction of									
Nitrate		–	–	–	±	–	±	–	–
Sulfate		–	–	–	–	–	–	–	–
Sulfite		–	–	nd	nd	±	–	–	–
Thiosulfate		–	–	nd	–	±	–	–	–
Major fatty acids‡		C_14 : 0_, C_16 : 0_, C_16:1_ω7*c*	C_14 : 0_, C_16 : 0_, C_16:1_ω7*c*	C_14 : 0_, C_16:1_ω7*c*	iso-C_15 : 0_, C_16 : 0_	nd	iso-C_15 : 0_, C_16 : 0_	nd	nd
									

*Beuscher and Andreesen [27] mention the inability of *E. angustum* to utilize any carbohydrates or purines from the list of compounds tested by Dürre *et al*. [16] but do not list their names.

†Hypoxanthine was utilized by *E. angustum* only in the presence of uric acid [27].

‡Fatty acid analyses of *C. acidurici*, *C. purinilyticum*, and *E. angustum* were carried out by the Identification Service of the DSMZ, Braunschweig, Germany, using Sherlock Microbial Identification System [58] of MIDI Inc. (Newark, DE, USA). Myristic acid C_14 : 0_ clearly predominated, making up at least 32 %, 25 %, and 36 %, respectively, of the total fatty acid content in these organisms.

Despite certain differences, the high degree of 16S rRNA gene sequence similarity, consistent clustering on 16S rRNA gene-based and protein-based trees (Figs 1, 2 and S2), and the similar metabolic properties justify unification of *C. acidurici*, *C. purinilyticum* and *E. angustum* into a higher-level taxon. It is important to note that *Tissierella*- and *Peptoniphilus*-containing clusters on both 16S rRNA gene-based and protein-based trees (Figs 1 and 2) correspond to family-level groupings, *Tissierellaceae* and *Peptoniphilaceae*, respectively [11, 12]. Thus, based on the available phenotypic, chemotaxonomic, and phylogenetic information, we propose the designation of *Gottschalkiaceae* fam. nov., to accommodate the genera *Gottschalkia* and *Andreesenia.* The novel family is easily distinguished by the ability of its members to use uric acid as the sole carbon and energy source and the predominance of myristic acid among the fatty acids. As a sister group of *Tissierellaceae* and *Peptoniphilaceae*, the proposed family *Gottschalkiaceae* could be tentatively assigned to the order *Tissierellales* within the class *Tissierellia* [12], although the high-order taxonomy of these organisms probably merits further study.

While the proposed family *Gottschalkiaceae* includes just three species with validly published names, representatives of this family appear to be widespread in nature. In their original description of *C. acidurici*, Barker and Beck [29] mentioned isolating very similar uric acid-degrading anaerobic bacteria from ten different soil samples from various places in California. They also isolated similar organisms from San Francisco bay mud and from sandy soil collected near Provo, Utah, and stated ‘No soil tested has ever failed to harbour the organisms’ [29]. Further, they found anaerobic uric acid-degrading bacteria in fecal material of the yellow-shafted flicker (*Colaptes auratus auratus*), an observation in line with uric acid being ‘the main nitrogenous end product of avian metabolism, which may be decomposed mainly by bacteria of this type’ [29].

Accordingly, a search of metagenomic sequence data identified *C. acidurici*-related 16S rRNA gene sequences in samples taken from a variety of habitats all over the world. These include, among others, the uncultured clones SBXY_753 and MAT-CR-P2-F02, collected from hypersaline microbial mats in the Guerrero Negro lagoon in Mexico [30] and in the Candeleria lagoon in Cabo Rojo, Puerto Rico [31], respectively; clone FGL12_B55 from an anoxygenic phototrophic community in Fayetteville Green Lake in upstate New York [32], and clone Q31013 from an intertidal sediment along the coast of Qinhuangdao in PR China [33] (Fig. 1). Metagenomic sequencing also revealed the presence of the uncultured clones, closely related to *C. acidurici*, *C. purinilyticum* and/or *E. angustum*, in ornithogenic soils of the Ross Sea region and King George Island in Antarctica, which form on land under the rookeries of Adélie penguins (e.g. clone 9/1/8C on Fig. 1) and Chinstrap and Gentoo penguins [34, 35]. This correlates with the finding of a closely related clone 1219A (GenBank accession no. FJ393497) in the fecal flora of Adélie penguins [36]. Finally, although not shown on Fig 1, 16S rRNA gene sequences falling within the radiation of the proposed family *Gottschalkiaceae* have been amplified from the samples taken from *Artemia*-associated microbiota in the solar salterns of Eilat, Israel [37], bovine mastitis milk [38], and anaerobic digesters treating poultry litter [39, 40], and other sources (see the https://www.arb-silva.de/browser/ssu-128/HE582772/ entry in the silva database [41] for more examples). These findings indicate the widespread distribution of *Gottschalkiaceae*-related organisms in both terrestrial and aquatic habitats, most likely due to their ability to metabolize avian-produced uric acid, as originally proposed by Barker and Beck [29].

## Description of *Gottschalkia* gen. nov.

*Gottschalkia* (Gott.schal′ki.a. N.L. fem. dim. n. *Gottschalkia* named after Professor Dr Gerhard Gottschalk in recognition of his important contributions to the studies of Clostridia).

Gram-stain-positive, obligately anaerobic, straight or slightly curved rods, 0.5–1.5×2.5–10 µm. Motile by means of lateral flagella. Growth occurs from 18–19 °C and up to 37–42 °C. The optimum temperature for growth is 30–37 °C. The pH range for growth is from 6.5 to 7.0 and up to 9.0; the optimum pH for growth is between 7.5 and 8.1. Form spores that are round to oval and terminal or subterminal. Chemoorganotrophs that require purines for growth, but do not utilize carbohydrates and most amino acids. In the presence of 0.1 % (w/v) yeast extract, can grow using uric acid as the sole carbon and energy source. Can also utilize guanine, purine, 2-hydroxypurine, xanthine and hypoxanthine. Major products of metabolism are acetate, formate, CO_2_ and NH_3_. Oxidase-, catalase-, lipase- and urease-negative. Nitrate and sulfate are not reduced. Cell walls contain *meso*-diaminopimelate. Isolated from soil, marine and freshwater sources and avian droppings.

The type species is *Gottschalkia acidurici* [basonym *Clostridiumacidurici* (Barker 1938) Approved List 1980]. The G+C content of the chromosomal DNA ranges from 28 to 30 mol%.

## Description of *Gottschalkia acidurici* comb. nov.

A.ci.du′ri.ci. N.L. gen. n. adj. *acidurici* of uric acid, referring to the preferred carbon source.

Basonym: *Clostridiumacidurici* (Liebert 1909) [29] (Approved List 1980).

The description of *Gottschalkia acidurici* is identical to that proposed for *Clostridiumacidurici* [2, 29, 42]. In addition to those described for the genus, has the following properties. Capable of growing in a salt medium containing 0.3 % (w/v) uric acid as the sole source of carbon, energy and nitrogen [42]. On an enrichment medium containing uric acid, forms whitish colonies 1–2 mm in diameter with irregular edges. Forms terminally located oval spores (0.9×1.1 µm in size) that cause a swelling of the cell.

The type strain *G. acidurici* 9a^T^(=ATCC 7906^T^=DSM 604^T^) was isolated from garden soil in California [29]. Its complete genome sequence [18] is available in GenBank under the accession no. CP003326. The G+C content of the genome is 29.9 mol% (27.8 % by the thermal denaturation method).

## Description of *Gottschalkia* *purinilytica* comb. nov.

Pu.ri.ni.ly′ti.ca. N.L. fem. adj. *purinilytica* lysing the purine ring.

Basonym: *Clostridiumpurinilyticum* Dürre, Andersch and Andreesen 1981.

The description of *Gottschalkia* *purinilytica* is identical to that for *Clostridiumpurinilyticum* [2, 16]. In addition to those described for the genus, has the following properties. Forms spherical terminally located endospores (0.8 to 1.2 µm in size) that result in swollen cells. Requires selenium compounds and thiamine for growth. Can use adenine, adenosine, inosine, or xanthosine as the sole source of carbon and energy. In the presence of purines, is able to utilize glycine, formiminoglycine, benzoylglycine, glycyl-glycine, glycyl-glycyl-glycine and glycyl-leucine.

The type strain WA-1^T^(=ATCC 33906^T^=DSM 1384^T^) was isolated from farm soil containing chicken manure in Bovenden-Eddigehausen, Germany [16]. 43The G+C content of the genome is 28.8 % [43].

## Description of ***Andreesenia*** **gen. nov.**

*Andreesenia* (An.dree.se′ni.a. N.L. fem. n. *Andreesenia* named after Professor Dr Jan Andreesen in recognition of his contributions to the studies of Clostridia).

Strictly anaerobic obligately purinolytic, Gram-stain-positive, non-spore-forming straight rods, 1.0–1.5×3–7 µm. Growth occurs from 18 to 45 °C (optimum temperature is 30–37 °C). The pH range for growth is from 6.5 to 10.0 (the optimum pH is between 7.5 and 8.5). In the presence of 0.1 % (w/v) yeast extract, can grow using uric acid as the sole carbon and energy source. Do not utilize carbohydrates, alcohols, amino acids, or organic acids. Do not grow on milk or chopped meat medium. The major products of metabolism are acetate, formate, CO_2_ and NH_3_. Oxidase-, catalase-, lipase- and urease-negative. Nitrate and sulfate are not reduced. Cell walls contain *meso*-diaminopimelate. Can be isolated from sewage, hypersaline microbial mats and avian droppings.

The type species is *Andreesenia angusta* (basonym *Eubacterium angustum* Beuscher and Andreesen 1985).

## Description of *Andreesenia angusta* comb. nov.

*An.gus′ta*. L. fem. adj. *angusta*, restricted, referring to the narrow substrate range.

Basonym: *Eubacterium angustum* Beuscher and Andreesen 1985.

The description of *Andreesenia angusta* is identical to that for *Eubacterium angustum* [27, 44]. In addition to those described for the genus, has the following properties. Non-motile but produces lateral flagella. Requires thiamine for growth, but does not require selenium, tungstate or molybdate. Nutritionally restricted to grow only on uric acid, guanine, or xanthine; in the presence of uric acid, can utilize hypoxanthine. Cells can grow in the presence of 2 % (w/v) bile extract. Colonies are nonpigmented, flat, circular, and 0.5–1.5 mm in diameter. Myristic (tetradecanoic) acid C_14 : 0_ makes up more than 36 mol% of all fatty acids. A draft genome sequence of the type strain has been deposited in the GenBank with the accession no. MKIE00000000 [28].

The type strain MK-1^T^(=ATCC 43737^T^=DSM 1989^T^) was isolated from sewage plant sludge in Göttingen, Germany [27]. The G+C content of the genome is 43.6 % (40.3 mol% by the thermal denaturation method).

## Description of *Gottschalkiaceae* fam. nov.

*Gottschalkiaceae* (Gott.schal.ki.a.ce′ae. N.L. fem. dim. n. *Gottschalkia* type genus of the family; L. suff. –*aceae* ending to denote a family; N.L. fem. pl. n. *Gottschalkiaceae* the family of the genus *Gottschalkia*).

Strictly anaerobic bacteria that can only grow by metabolizing purines. Gram-stain-positive, straight or slightly curved rods, 0.5–1.5×2–10 µm. Produces lateral and subterminal flagella. Growth occurs from 18–19 °C to 37–42 °C, the optimum growth temperature is 30–37 °C. The pH range is from 6.5 to 7.0 to 9.0, with the optimum pH between 7.5 and 8.2. May be spore-forming or asporogenous. In the presence of 0.1 % (w/v) yeast extract, can grow using uric acid, guanine, or xanthine as the sole carbon and energy sources; some representatives may also utilize other purines. Do not utilize carbohydrates and most amino acids; in the presence of purines, may use glycine, serine, or glycine-containing peptides. Major products of metabolism are acetate, formate, CO_2_ and NH_3_. Oxidase-, catalase-, lipase- and urease-negative. Nitrate and sulfate are not reduced. Cell walls contain *meso*-diaminopimelate. Predominant fatty acids are C_14 : 0_ and C_16 : 1_. Often associated with avian droppings and can be isolated from soil, and aquatic marine and freshwater sources.

The family includes the genera *Gottschalkia* and *Andreesenia*. The type genus is the genus *Gottschalkia*. The G+C content of the chromosomal DNA ranges from 28 to 44 mol%.

## Supplementary Data

243 Supplementary File 1
