# Supplementary material for: Proposal for the reclassification of obligately purine-fermenting bacteria Clostridium acidurici (Barker 1938) and Clostridium purinilyticum (Dürre et al. 1981) as Gottschalkia acidurici gen. nov. comb. nov. and Gottschalkia purinilytica comb. nov. and of Eubacterium angustum (Beuscher and Andreesen 1985) as Andreesenia angusta gen. nov. comb. nov. in the family Gottschalkiaceae fam. nov
Source: Int J Syst Evol Microbiol. 2017 Aug 15;67(8):2711–9. doi: 10.1099/ijsem.0.002008 (PMC5737214; doi:10.1099/ijsem.0.002008)
Supplement: Supplementary File 1 [file ijsem-67-2711-s001.pdf]

Supplementary Material for

Proposal for reclassification of obligately purine-fermenting bacteria *Clostridium acidurici* (Liebert 1909) Barker & Beck 1942 and *Clostridium purinilyticum* (Dürre *et al.* 1981) as *Gottschalkia acidurici* gen. nov. comb. nov. and *Gottschalkia purinilytica* comb. nov. and of *Eubacterium angustum* (Beuscher & Andreesen 1985) as *Andreesenia angusta* gen. nov. comb. nov. in the family *Gottschalkiaceae* fam. nov.

by

Anja Poehlein, Natalya Yutin, Rolf Daniel and Michael Y. Galperin

SUPPLEMENTARY METHODS

1. Construction of 16S rRNA gene-based trees

The 16S rRNA gene trees of *C. acidurici*, *C. purinilyticum*, and *E. angustum* and their neighbors from clusters I, XI, XII, and XIII were constructed using the neighbor-joining (Fig. 1) and maximum likelihood methods (see Fig. S1 below). The 16S rRNA gene sequences of the type strains were obtained either from GenBank or from the NCBI RefSeq Targeted Loci project (Federhen, 2015), as specified in the List of Prokaryotic Names with Standing in Nomenclature (Parte, 2014). Specifically, for the 16S rRNA gene sequence of the *C. acidurici* type strain 9a^T (=DSM 604^T), we have chosen the RefSeq entry NR_117601, which is 100% identical to three out of six 16S rRNAs encoded in its complete genome (GenBank accession no. CP003326, positions 11128-12640, 2471929-2473441, and 3033096-3034608) and 99% identical to three other rRNAs (positions 992015-993528, 1003103-1004615, and 3020878-3022390). It is also 100% identical but longer than GenBank accession HE582772 and lacks the ambiguous nucleotide assignments found in the accession M59084, the current entry in the LPSN (Parte, 2014). For the 16S rRNA gene sequence of the *C. purinilyticum* type strain DSM 1384^T (=ATCC 33906^T), we have chosen GenBank accession FR749894, which is 100% identical to the RefSeq entry NR_117121, 99% identical to four partial 16S rRNA genes encoded in its current genome assembly (GenBank accessions LGSS01000012, LGSS01000054, LGSS01000056, and LGSS01000064), and contains fewer ambiguous nucleotides than GenBank accession M60491. For *E. angustum* type strain DSM 1989^T

(=ATCC 43737^T), we used its canonical 16S rRNA gene sequence (GenBank accession L34612), which is 99% identical to the gene found in its recently sequenced genome (Poehlein *et al.*, 2017), GenBank accession MKIE00000000. Sequences were aligned with ClustalW (Thompson *et al.*, 1994), as implemented in the MEGA7 software suite (Kumar *et al.*, 2016), and the neighbor-joining and maximum likelihood trees were constructed using MEGA7.

2. Construction of the ribosomal proteins-based tree

The ribosomal proteins-based phylogenetic tree (Fig. 2) was constructed from a concatenated alignment of 50 widespread ribosomal proteins (L1–L7, L9–L11, L13–L24, L27–L29, L31–L36 and S2–S20), as described earlier (Yutin *et al.*, 2012; Yutin & Galperin, 2013). Protein sequences were taken from selected organisms with completely or partially sequenced genomes, where available (see Table S1 below). The missing 50S ribosomal protein L9 of *C. acidurici* was translated from the genome entry CP003326:3004005..3004453 by correcting a frameshift in the middle of its open reading frame. In those cases when the respective genomes had two *rpsD* genes encoding two different S4 proteins, we selected the protein encoded in the *rps* operon, between *rpsK* and *rpoA* genes (the topology of the final tree did not change upon inclusion or excluson of S4 proteins). The missing 50S ribosomal proteins L36 from *Sporanaerobacter acetigenes* DSM 13106^T, *Tepidimicrobium xylanilyticum* DSM 23310^T, *Tissierella praeacuta* DSM 18095^T, and *Tissierella creatinophila* DSM 6911^T have been translated from the respective genomic sequences; all of them proved identical to the NCBI RefSeq entry WP_041701799.1. Ribosomal protein sequences were aligned by MUSCLE (Edgar, 2004), these alignments were concatenated, and the resulting 6,222-position alignment was used to construct a phylogenetic tree using the PhyML program (Guindon *et al.*, 2010).

3. Trees for Individual ribosomal proteins

Approximately maximum-likelihood trees for individual ribosomal proteins were constructed using FastTree2 (Price *et al.*, 2010). Examination of those trees showed that in 37 out of 50 trees *C. acidurici*, *C. purinilyticum*, and *E. angustum* were grouped together in a single cluster; in 28 of these trees, those clusters had local support values of at least 70% (data not shown).

4. Construction of the RpoB and GyrB trees

The sequences of DNA-directed RNA polymerase beta subunit (RpoB) and DNA gyrase subunit B (GyrB) from various members of clostridial clusters I, XI, XII, and XII with completely or partially sequenced genomes (Table S1), were extracted from the NCBI protein database. The sequences were aligned with MUSCLE (Edgar, 2004), as implemented in the MEGA7 software suite (Kumar *et al.*, 2016). The evolutionary history was inferred by using the maximum likelihood method based on the JTT matrix-based model (Jones *et al.*, 1992) and the trees with the highest log likelihood are shown in Fig. S2 (a) and (b). The percentage of trees in which the associated taxa clustered together is shown next to the branches. Initial tree(s) for the heuristic search were obtained automatically by applying neighbor-join and BioNJ algorithms to a matrix of pairwise distances estimated using a JTT model, and then selecting the topology with superior log likelihood value. The tree is drawn to scale, with branch lengths measured in the number of substitutions per site. In both cases, the analysis involved 23 amino acid sequences. All positions containing gaps and missing data were eliminated. There were a total of 1159 positions in the final dataset for RpoA and 627 positions for GyrB. Evolutionary analyses were conducted in MEGA7 (Kumar *et al.*, 2016).

5. Search of metagenomic sequence data

Searches for *C. acidurici*-related 16S rRNA gene sequences were performed using BLASTn against the NCBI's nucleotide collection and the whole-genome shotgun contigs (WGS) database. The high-scoring hits were individually tested by reverse BLAST against the NCBI's database of 16S rRNA sequences (to ensure that they fall within the radiation of the proposed family *Gottschalkiaceae*) and checked for the presence of an associated publication and/or information on sampling location. We also checked selected entries from *Clostridiales* Family XI in the SILVA database (https://www.arb-silva.de/browser/ssu-128/HE582772) (Yilmaz *et al.*, 2014).

Table S1. Source data for the phylogenetic trees

Organism name[a,b]	GenBank accession number		Genome reference or sequencing center
	16S rRNA	Genome	
**Proposed family *Gottschalkiaceae***			
**Proposed *Gottschalkia* spp.**			
Clostridium acidurici DSM 604^{T}	NR_117601	CP003326	Hartwich *et al.* (2012)
Clostridium purinilyticum DSM 1384^{T}	FR749894	LGSS00000000	Poehlein *et al.* (2015a)
**Proposed *Andreesenia* sp.**			
Eubacterium angustum DSM 1989^{T}	L34612	MKIE00000000	Poehlein *et al.* (2017)
**Clostridial cluster I (family *Clostridiaceae*)**			
Clostridium butyricum DSM 10702^{T}	AJ458420	AQQF00000000	Xin *et al.* (2013)
Clostridium cylindrosporum DSM 605^{T}	Y18179	LFVU00000000	Poehlein *et al.* (2015b)
**Clostridial cluster XI (family *Peptostreptococcaceae*)**			
Clostridioides difficile[b] DSM 1296^{T}	AB075770	CP011968	Riedel *et al.* (2015)
Acetoanaerobium sticklandii[b] DSM 519^{T}	NR_102880	FP565809	Fonknechten *et al.* (2010)
Paeniclostridium sordellii ATCC 9714^{T}	AB075771	APWR00000000	Sirigi Reddy *et al.* (2013)
Paraclostridium bifermentans ATCC 638^{T}	AB075769	AVNC00000000	Univ. Maryland Sch. of Medicine, unpublished
Peptoclostridium litorale[b] DSM 5388^{T}	X77845	JJMM00000000	Poehlein *et al.* (2014)
Peptostreptococcus anaerobius DSM 2949^{T}	AY326462	ARMA00000000	DOE-JGI, unpublished
**Clostridial cluster XII (family *Tissierellaceae*)**			
Anaerosalibacter massiliensis[b] (*Anaerosalibacter* sp.) ND1^{T} = DSM 27308	HG315673	CCEZ00000000	Dione *et al.* (2016)
[*Clostridium*] *ultunense*[b] DSM 10521^{T}	GQ461825	AZSU00000000	Wei *et al.* (2014)
Soehngenia saccharolytica DSM 12858^{T}	GQ461828	n/a[c]	n/a
Tissierella creatinophila DSM 6911^{T}	GQ461823	LTDM00000000	Nacke *et al.* (2017)
Tissierella praeacuta ATCC 25539^{T}	X80833	FQTY01000000	DOE-JGI, unpublished
**Clostridial cluster XIII (family *Peptoniphilaceae*)**			
Anaerococcus prevotii DSM 20548^{T}	NR_074575	CP001708	Labutti *et al.* (2009)
Anaerosphaera aminiphila DSM 21120^{T}	AB298735	FQXI00000000	DOE-JGI, unpublished
Finegoldia magna ATCC 29328^{T}	NR_074677	AP008971	Goto *et al.* (2008)
Helcococcus kunzii ATCC 51366^{T}	X69837	AGEI00000000	Broad Inst., unpublished

Murdochiella asaccharolytica ATCC BAA-1631[T]	EU483153	n/a[c]	n/a
Parvimonas micra ATCC 33270[T]	AY323523	ABEE00000000	Washington Univ., St. Louis, unpublished
Peptoniphilus lacrimalis DSM 7455[T]	AF542230	ARKX00000000	DOE-JGI, unpublished
**Unclassified *Tissierellia***			
Sporanaerobacter acetigenes DSM 13106[T]	AF358114	FQXR00000000	DOE-JGI, unpublished
Tepidimicrobium xylanilyticum DSM 23310[T]	EF522948	FNNG01000000	DOE-JGI, unpublished

[a] – As of February 1st, 2017. Assignments to *Clostridium* clusters are from Collins *et al*. (1994), family assignments are from Alauzet *et al*. (2014), Galperin *et al*. (2016), and Johnson *et al*. (2014).

[b] – *Clostridioides difficile, Acetoanaerobium sticklandii*, and *Peptoclostridium litorale* are recently renamed former *Clostridium* species (Galperin *et al.,* 2016; Lawson *et al.,* 2016). [*Clostridium*] *ultunense* is a misclassified but validly described organism. The name *Anaerosalibacter massiliensis* has not been validly published at the time of this writing.

[c] – *Soehngenia saccharolytica* and *Murdochiella asaccharolytica* have been included in the 16S rRNA gene-based trees (Fig. 1 and S1) but not in protein-based trees (Fig. 2 and S2). On protein trees, "*Murdochiella massiliensis*" strain SIT12 and *Murdochiella* sp. Marseille-P2341, neither of which has been validly described, clustered with *Helcococcus kunzii* ATCC 51366[T].

Table S2. Sporulation genes in *C. acidurici*, *C. purinilyticum* and *E. angustum*[a]

Gene name (*B. subtilis*)	*Clostridium acidurici* ORF	BLAST E-value	*Clostridium purinilyticum* ORF	BLAST E-value	*Eubacterium angustum* ORF	BLAST E-value
spo0A	Curi_c13920	1.4E-119	CLPU_17c00380	3.0E-93	EUAN_05540	6.0E-15
sigE	Curi_c13090	3.1E-118	CLPU_1c00780	1.0E-87	EUAN_11380	1.0E-12
sigF	Curi_c10310	1.5E-90	CLPU_3c00440	4.0E-72	EUAN_23280	2.0E-39
spoIIQ	Curi_c28430	6.9E-15	CLPU_32c00040	9.0E-11	EUAN_08450	1.0E-10
spoIIIE	Curi_c15450	1.0E-200	CLPU_5c01850	1.0E-155	EUAN_05950	2.0E-154
spoIIIJ	Curi_c29550	2.3E-30	CLPU_19c00300	7.0E-21	EUAN_23410	5.0E-20
spoVB	Curi_c07380	1.4E-53	CLPU_12c00810	2.0E-41	EUAN_17640	1.0E-48
spoVC	Curi_c23840	2.4E-48	CLPU_12c00770	8.0E-45	EUAN_17670	8.0E-42
spoVD	Curi_c12960	2.5E-124	CLPU_1c00910	5.0E-127	EUAN_08190	1.0E-102
spoVE	Curi_c13000	4.7E-95	CLPU_1c00870	1.0E-74	EUAN_08150	2.0E-64
spoVK	Curi_c15210	3.2E-99	CLPU_5c02260	2.0E-82	EUAN_13590	6.0E-51
spoVS	Curi_c15390	2.0E-54	CLPU_5c01920	3.0E-34	EUAN_06010	2.0E-34
spoVT	Curi_c23810	7.1E-71	CLPU_12c00800	7.0E-61	EUAN_21930	8.0E-14
bofA	Curi_c01030	2.7E-04	CLPU_15c00040	6.1E-02	EUAN_20560	5.0E-04
dapA	Curi_c11790	2.9E-90	CLPU_1c01850	4.0E-71	EUAN_18870	6.0E-64
dapB	Curi_c11780	8.3E-24	CLPU_1c01860	5.0E-20	EUAN_18860	4.0E-16
gerM	Curi_c03250	1.8E-20	CLPU_20c00080	6.0E-23	EUAN_16680	2.0E-05
spmA	Curi_c01200	1.0E-42	CLPU_2c02710	7.0E-43	–	–
spmB	Curi_c01210	2.0E-41	CLPU_2c02700	3.0E-44	–	–
spoIID	Curi_c02090	8.0E-50	CLPU_2c01570	2.0E-50	–	–
spoIIM	Curi_c14030	3.0E-29	CLPU_17c00460	5.0E-25	–	–
spoIIP	Curi_c18350	3.0E-24	CLPU_3c01440	2.0E-18	–	–
spoIIR	Curi_c01580	8.0E-33	CLPU_2c02290	8.0E-35	–	–
spoIIIAA	Curi_c13720	2.0E-66	CLPU_17c00160	2.0E-70	–	–
spoIIIAB	Curi_c13730	1.0E-21	CLPU_17c00170	1.0E-13	–	–
spoIIIAC	Curi_c13740	8.0E-08	CLPU_17c00180	1.0E-12	–	–
spoIIIAD	Curi_c13750	5.0E-26	CLPU_17c00190	9.0E-32	–	–
spoIIIAE	Curi_c13760	2.0E-53	CLPU_17c00200	1.0E-55	–	–
spoIIIAF	Curi_c13770	9.0E-05	CLPU_10c00130	2.0E-14	–	–
spoIIIAG	Curi_c13780	1.0E-08	CLPU_17c00220	2.0E-08	–	–
spoIIIAH	Curi_c13790	3.0E-05	CLPU_17c00230	2.0E-07	–	–
spoIIID	Curi_c02110	1.0E-25	CLPU_2c01550	7.0E-26	–	–
spoIVA	Curi_c17050	8.0E-149	CLPU_5c00240	5.0E-152	–	–
spoIVB	Curi_c13910	1.0E-82	CLPU_17c00370	7.0E-92	–	–
spoVAC	Curi_c10320	2.0E-27	CLPU_3c00390	1.0E-26	–	–
spoVAD	Curi_c10330	2.0E-60	CLPU_3c00380	2.0E-78	–	–
spoVAEB	Curi_c10340	3.0E-17	CLPU_3c00370	2.0E-18	–	–
spoVAF	Curi_c18660	3.0E-100	CLPU_3c00350	6.0E-113	–	–
spoVG	Curi_c23870	1.0E-27	CLPU_12c00740	2.0E-28	–	–

[a] – Best hits and expectation values of these hits being obtained solely by chance in the BLASTp searches of the protein databases of *C. acidurici*, *C. purinilyticum*, and E. *angustum* with proteins from *Bacillus subtilis* used as queries. A dash indicates the absence of the respective gene (protein) in *E. angustum.*

References

Alauzet, C., Marchandin, H., Courtin, P., Mory, F., Lemee, L., Pons, J. L., Chapot-Chartier, M. P., Lozniewski, A. & Jumas-Bilak, E. (2014). Multilocus analysis reveals diversity in the genus *Tissierella*: description of *Tissierella carlieri* sp. nov. in the new class *Tissierellia* classis nov. *Syst Appl Microbiol* **37**: 23-34.

Collins, M. D., Lawson, P. A., Willems, A., Cordoba, J. J., Fernandez-Garayzabal, J., Garcia, P., Cai, J., Hippe, H. & Farrow, J. A. E. (1994). The phylogeny of the genus *Clostridium*: proposal of five new genera and eleven new species combinations. *Int J Syst Bacteriol* **44**: 812-826.

Dione, N., Sankar, S. A., Lagier, J. C., Khelaifia, S., Michele, C., Armstrong, N., Richez, M., Abrahao, J., Raoult, D. & other authors (2016). Genome sequence and description of *Anaerosalibacter massiliensis* sp. nov. *New Microbes New Infect* **10**: 66-76.

Edgar, R. C. (2004). MUSCLE: multiple sequence alignment with high accuracy and high throughput. *Nucleic Acids Res* **32**: 1792-1797.

Federhen, S. (2015). Type material in the NCBI Taxonomy Database. *Nucleic Acids Res* **43**: D1086-D1098.

Fonknechten, N., Chaussonnerie, S., Tricot, S., Lajus, A., Andreesen, J. R., Perchat, N., Pelletier, E., Gouyvenoux, M., Barbe, V. & other authors (2010). *Clostridium sticklandii*, a specialist in amino acid degradation: revisiting its metabolism through its genome sequence. *BMC Genomics* **11**: 555.

Galperin, M. Y., Brover, V., Tolstoy, I. & Yutin, N. (2016). Phylogenomic analysis of the family *Peptostreptococcaceae* (*Clostridium* cluster XI) and proposal for reclassification of *Clostridium litorale* (Fendrich *et al*. 1991) and *Eubacterium acidaminophilum* (Zindel *et al*. 1989) as *Peptoclostridium litorale* gen. nov. comb. nov. and *Peptoclostridium acidaminophilum* comb. nov. *Int J Syst Evol Microbiol* **66**: 5506–5513.

Goto, T., Yamashita, A., Hirakawa, H., Matsutani, M., Todo, K., Ohshima, K., Toh, H., Miyamoto, K., Kuhara, S. & other authors (2008). Complete genome sequence of *Finegoldia magna*, an anaerobic opportunistic pathogen. *DNA Res* **15**: 39-47.

Guindon, S., Dufayard, J. F., Lefort, V., Anisimova, M., Hordijk, W. & Gascuel, O. (2010). New algorithms and methods to estimate maximum-likelihood phylogenies: assessing the performance of PhyML 3.0. *Syst Biol* **59**: 307-321.

Hartwich, K., Poehlein, A. & Daniel, R. (2012). The purine-utilizing bacterium *Clostridium acidurici* 9a: a genome-guided metabolic reconsideration. *PLoS One* **7**: e51662.

Johnson, C. N., Whitehead, T. R., Cotta, M. A., Rhoades, R. E. & Lawson, P. A. (2014). *Peptoniphilus stercorisuis* sp. nov., isolated from a swine manure storage tank and description of *Peptoniphilaceae* fam. nov. *Int J Syst Evol Microbiol* **64**: 3538-3545.

Jones, D. T., Taylor, W. R. & Thornton, J. M. (1992). The rapid generation of mutation data matrices from protein sequences. *Comput Appl Biosci* **8**: 275-282.

Kumar, S., Stecher, G. & Tamura, K. (2016). MEGA7: Molecular Evolutionary Genetics Analysis version 7.0 for bigger datasets. *Mol Biol Evol* **33**: 1870-1874.

Labutti, K., Pukall, R., Steenblock, K., Glavina Del Rio, T., Tice, H., Copeland, A., Cheng, J. F., Lucas, S., Chen, F. & other authors (2009). Complete genome sequence of *Anaerococcus prevotii* type strain (PC1). *Stand Genomic Sci* **1**: 159-165.

Lawson, P. A., Citron, D. M., Tyrrell, K. L. & Finegold, S. M. (2016). Reclassification of *Clostridium difficile* as *Clostridioides difficile* (Hall and O'Toole 1935) Prevot 1938. *Anaerobe* **40**: 95-99.

Nacke, H., Daniel, R. & Poehlein, A. (2017). Genome sequence of creatinine-fermenting *Tissierella creatinophila* strain KRE 4^T (DSM 6911). *Genome Announc* **5**: e00051-00017.

Parte, A. C. (2014). LPSN--list of prokaryotic names with standing in nomenclature. *Nucleic Acids Res* **42**: D613-D616.

Poehlein, A., Alghaithi, H. S., Chandran, L., Chibani, C. M., Davydova, E., Dhamotharan, K., Ge, W., Gutierrez-Gutierrez, D. A., Jagirdar, A. & other authors (2014). First insights into the genome of the amino acid-metabolizing bacterium *Clostridium litorale* DSM 5388. *Genome Announc* **2**: e00754-00714.

Poehlein, A., Bengelsdorf, F. R., Schiel-Bengelsdorf, B., Daniel, R. & Dürre, P. (2015a). Draft genome sequence of purine-degrading *Gottschalkia purinilyticum* (formerly *Clostridium purinilyticum*) WA1 (DSM 1384). *Genome Announc* **3**: e01088-01015.

Poehlein, A., Montoya Solano, J. D., Bengelsdorf, F. R., Schiel-Bengelsdorf, B., Daniel, R. & Dürre, P. (2015b). Draft genome sequence of purine-degrading *Clostridium cylindrosporum* HC-1 (DSM 605). *Genome Announc* **3**: e00917-00915.

Poehlein, A., Galperin, M. Y., Andreesen, J. R. & Daniel, R. (2017). Genome sequence of uric acid-fermenting *Eubacterium angustum* DSM 1989T (MK-1). *Genome Announc* **5**: e01439-01416.

Price, M. N., Dehal, P. S. & Arkin, A. P. (2010). FastTree 2--approximately maximum-likelihood trees for large alignments. *PLoS One* **5**: e9490.

Riedel, T., Bunk, B., Wittmann, J., Thurmer, A., Sproer, C., Gronow, S., Liesegang, H., Daniel, R. & Overmann, J. (2015). Complete genome sequence of the *Clostridium difficile* type strain DSM 1296^T. *Genome Announc* **3**: e01186-01115.

Sirigi Reddy, A. R., Girinathan, B. P., Zapotocny, R. & Govind, R. (2013). Identification and characterization of *Clostridium sordellii* toxin gene regulator. *J Bacteriol* **195**: 4246-4254.

Tamura, K. & Nei, M. (1993). Estimation of the number of nucleotide substitutions in the control region of mitochondrial DNA in humans and chimpanzees. *Mol Biol Evol* **10**: 512-526.

Thompson, J. D., Higgins, D. G. & Gibson, T. J. (1994). CLUSTAL W: improving the sensitivity of progressive multiple sequence alignment through sequence weighting, position-specific gap penalties and weight matrix choice. *Nucleic Acids Res* **22**: 4673-4680.

Wei, Y., Zhou, H., Zhang, L., Zhang, J., Wang, Y., Wang, S., Zhou, Z. & Yan, X. (2014). Draft genome sequence of *Clostridium ultunense* strain BS (DSMZ 10521), recovered from a mixed culture. *Genome Announc* **2**: e01269-01213.

Xin, B., Tao, F., Wang, Y., Gao, C., Ma, C. & Xu, P. (2013). Genome sequence of *Clostridium butyricum* strain DSM 10702, a promising producer of biofuels and biochemicals. *Genome Announc* **1**: e00563-00513.

Yilmaz, P., Parfrey, L. W., Yarza, P., Gerken, J., Pruesse, E., Quast, C., Schweer, T., Peplies, J., Ludwig, W. & other authors (2014). The SILVA and "All-species Living Tree Project (LTP)" taxonomic frameworks. *Nucleic Acids Res* **42**: D643-D648.

Yutin, N., Puigbo, P., Koonin, E. V. & Wolf, Y. I. (2012). Phylogenomics of prokaryotic ribosomal proteins. *PLoS One* **7**: e36972.

Yutin, N. & Galperin, M. Y. (2013). A genomic update on clostridial phylogeny: Gram-negative spore formers and other misplaced clostridia. *Environ Microbiol* **15**: 2631-2641.

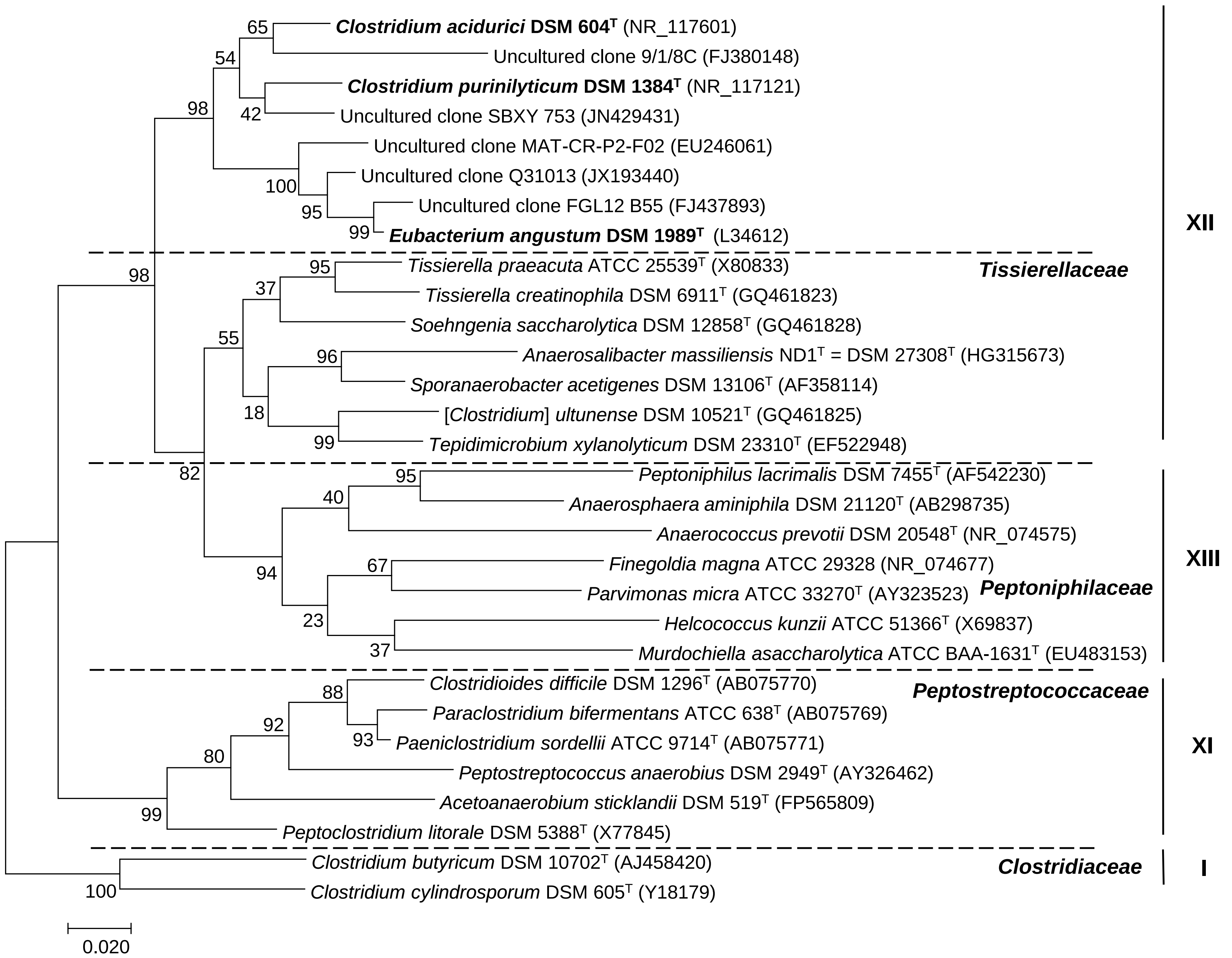
Clostridium acidurici DSM 604T (NR_117601)
Uncultured clone 9/1/8C (FJ380148)
Clostridium purinilyticum DSM 1384T (NR_117121)
Uncultured clone SBXY 753 (JN429431)
Uncultured clone MAT-CR-P2-F02 (EU246061)
Uncultured clone Q31013 (JX193440)
Uncultured clone FGL12 B55 (FJ437893)
Eubacterium angustum DSM 1989T (L34612)
Tissierella praeacuta ATCC 25539T (X80833)
Tissierella creatinophila DSM 6911T (GQ461823)
Soehngenia saccharolytica DSM 12858T (GQ461828)
Anaerosalibacter massiliensis ND1T = DSM 27308T (HG315673)
Sporanaerobacter acetigenes DSM 13106T (AF358114)
[Clostridium] ultunense DSM 10521T (GQ461825)
Tepidimicrobium xylanolyticum DSM 23310T (EF522948)
Peptoniphilus lacrimalis DSM 7455T (AF542230)
Anaerosphaera aminiphila DSM 21120T (AB298735)
Anaerococcus prevotii DSM 20548T (NR_074575)
Finegoldia magna ATCC 29328 (NR_074677)
Parvimonas micra ATCC 33270T (AY323523)
Helcococcus kunzii ATCC 51366T (X69837)
Murdochiella asaccharolytica ATCC BAA-1631T (EU483153)
Clostridioides difficile DSM 1296T (AB075770)
Paraclostridium bifermentans ATCC 638T (AB075769)
Paeniclostridium sordellii ATCC 9714T (AB075771)
Peptostreptococcus anaerobius DSM 2949T (AY326462)
Acetoanaerobium sticklandii DSM 519T (FP565809)
Peptoclostridium litorale DSM 5388T (X77845)
Clostridium butyricum DSM 10702T (AJ458420)
Clostridium cylindrosporum DSM 605T (Y18179)
XII
Tissierellaceae
XIII
Peptoniphilaceae
Peptostreptococcaceae
XI
Clostridiaceae
I
0.020

Figure S1. 16S rRNA gene-based phylogenetic tree of proposed *Gottschalkiaceae* species, related organisms and uncultured metagenomic samples. Members of the proposed new genera *Gottschalkia* and *Andreesenia* are shown in bold. The sequences from type strains (indicated with ^{T}) were used and listed under their DSM accession numbers, where available. GenBank accession numbers are listed in parentheses. Roman numerals on the right indicate clostridial cluster assignments of Collins *et al*. (1994). The evolutionary history was inferred by using the maximum likelihood method based on the Tamura-Nei model (Tamura & Nei, 1993) as implemented in MEGA7 (Kumar *et al.*, 2016). The percentage of trees in which the associated taxa clustered together is shown next to the branches. The tree is drawn to scale, with branch lengths measured in the number of substitutions per site. All positions containing gaps and missing data were eliminated. There were a total of 1273 positions in the final dataset. The tree was rooted using sequences from *C. butyricum* and *C. cylindrosporum*, members of *Clostridium sensu stricto* (cluster I). The name *Anaerosalibacter massiliensis* has been effectively published (Dione et al., 2016) but not validly published at the time of this writing.

Fig. S2a

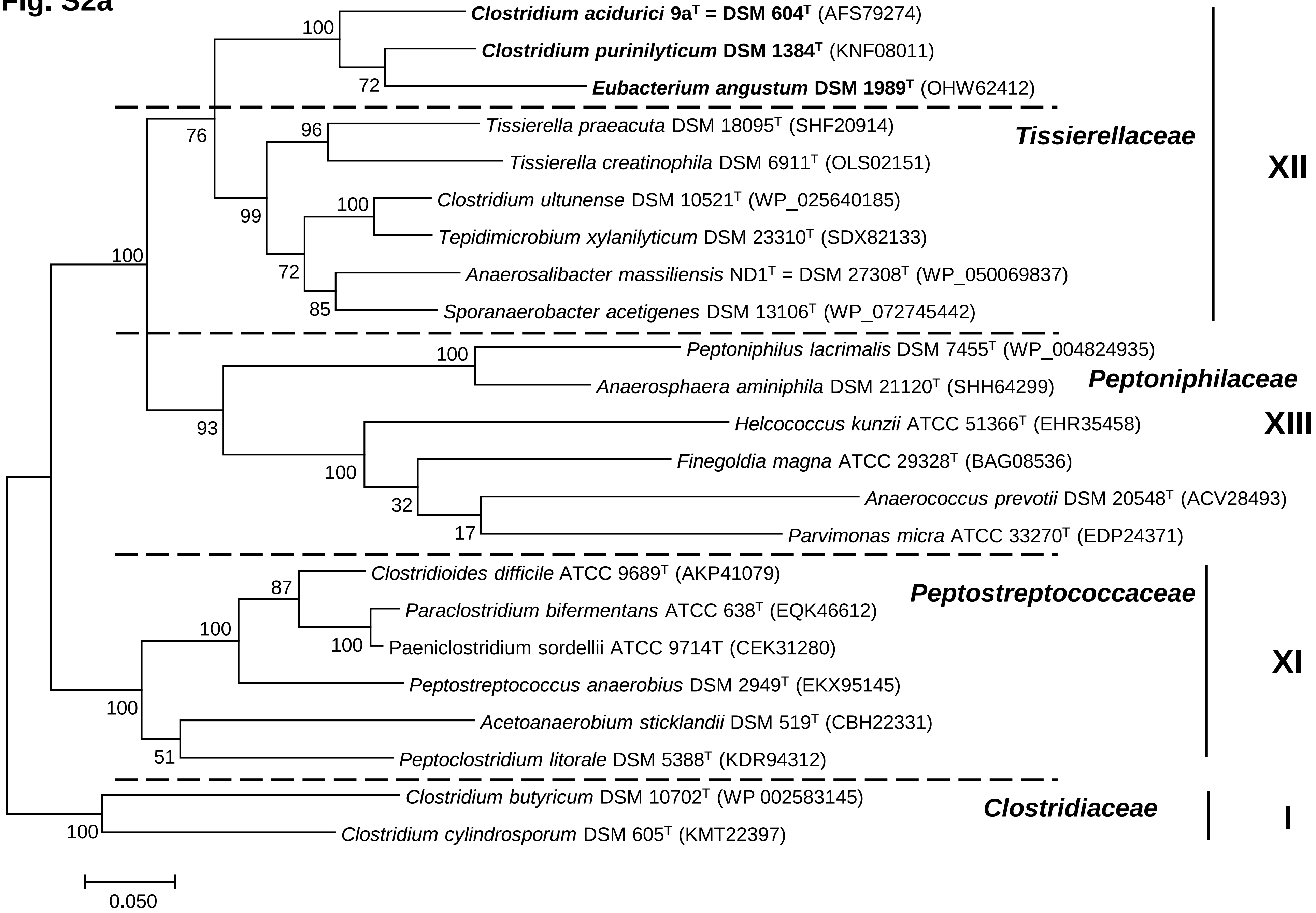

Clostridium acidurici 9aT = DSM 604T (AFS79274)
Clostridium purinilyticum DSM 1384T (KNF08011)
Eubacterium angustum DSM 1989T (OHW62412)
Tissierella praeacuta DSM 18095T (SHF20914)
Tissierella creatinophila DSM 6911T (OLS02151)
Clostridium ultunense DSM 10521T (WP_025640185)
Tepidimicrobium xylanilyticum DSM 23310T (SDX82133)
Anaerosalibacter massiliensis ND1T = DSM 27308T (WP_050069837)
Sporanaerobacter acetigenes DSM 13106T (WP_072745442)
Tissierellaceae
XII
Peptoniphilus lacrimalis DSM 7455T (WP_004824935)
Anaerosphaera aminiphila DSM 21120T (SHH64299)
Helcococcus kunzii ATCC 51366T (EHR35458)
Finegoldia magna ATCC 29328T (BAG08536)
Anaerococcus prevotii DSM 20548T (ACV28493)
Parvimonas micra ATCC 33270T (EDP24371)
Peptoniphilaceae
XIII
Clostridioides difficile ATCC 9689T (AKP41079)
Paraclostridium bifermentans ATCC 638T (EQK46612)
Paeniclostridium sordellii ATCC 9714T (CEK31280)
Peptostreptococcus anaerobius DSM 2949T (EKX95145)
Acetoanaerobium sticklandii DSM 519T (CBH22331)
Peptoclostridium litorale DSM 5388T (KDR94312)
Peptostreptococcaceae
XI
Clostridium butyricum DSM 10702T (WP 002583145)
Clostridium cylindrosporum DSM 605T (KMT22397)
Clostridiaceae
I
100
72
76
96
99
100
72
85
100
100
93
100
32
17
87
100
100
100
51
100
0.050

Fig. S2b

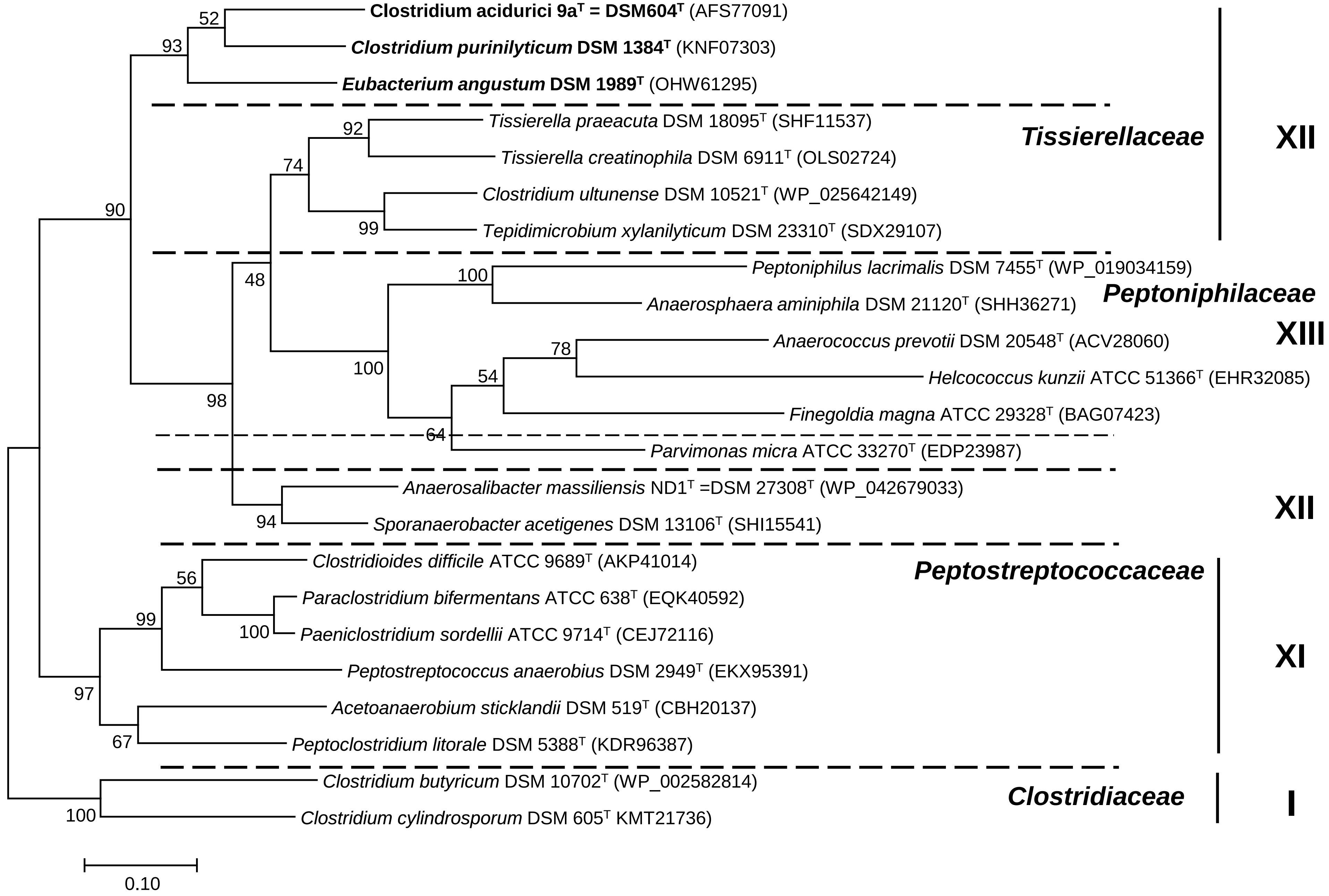

Clostridium acidurici 9aT = DSM604T (AFS77091)
Clostridium purinilyticum DSM 1384T (KNF07303)
Eubacterium angustum DSM 1989T (OHW61295)
Tissierella praeacuta DSM 18095T (SHF11537)
Tissierella creatinophila DSM 6911T (OLS02724)
Clostridium ultunense DSM 10521T (WP_025642149)
Tepidimicrobium xylanilyticum DSM 23310T (SDX29107)
Peptoniphilus lacrimalis DSM 7455T (WP_019034159)
Anaerosphaera aminiphila DSM 21120T (SHH36271)
Anaerococcus prevotii DSM 20548T (ACV28060)
Helcococcus kunzii ATCC 51366T (EHR32085)
Finegoldia magna ATCC 29328T (BAG07423)
Parvimonas micra ATCC 33270T (EDP23987)
Anaerosalibacter massiliensis ND1T =DSM 27308T (WP_042679033)
Sporanaerobacter acetigenes DSM 13106T (SHI15541)
Clostridioides difficile ATCC 9689T (AKP41014)
Paraclostridium bifermentans ATCC 638T (EQK40592)
Paeniclostridium sordellii ATCC 9714T (CEJ72116)
Peptostreptococcus anaerobius DSM 2949T (EKX95391)
Acetoanaerobium sticklandii DSM 519T (CBH20137)
Peptoclostridium litorale DSM 5388T (KDR96387)
Clostridium butyricum DSM 10702T (WP_002582814)
Clostridium cylindrosporum DSM 605T KMT21736)
Tissierellaceae
XII
Peptoniphilaceae
XIII
XII
Peptostreptococcaceae
XI
Clostridiaceae
I
52
93
92
74
99
90
100
48
100
78
54
98
64
94
56
99
100
97
67
100
0.10

Figure S2. Phylogenetic trees of RpoB (A) and GyrB (B) sequences from *Clostridium acidurici* and related organisms.

Members of the proposed family *Gottschalkiaceae* (genera *Gottschalkia* and *Andreesenia*) are shown in bold. The sequences were taken from the respective genome entries, listed in Table S1. The protein accession numbers in the NCBI protein database are shown in parentheses. The sequences from type strains (indicated with ^{T}) were used and listed under their DSM accession numbers, where available. Roman numerals on the right indicate clostridial cluster assignments of Collins *et al*. (1994). The dashed lines indicate the boundaries between the family *Tissereliaceae* (in cluster XII), *Peptonophilaceae* (cluster XIII), *Peptostreptococcaceae* (cluster XI) and *Clostridiaceae* (cluster I), see Alauzet *et al*. (2014), Johnson *et al*. (2014), and Galperin *et al*. (2016). The alignments were constructed using MUSCLE (Edgar, 2004) and the evolutionary history was inferred by using the maximum likelihood method based on the Tamura-Nei model (Tamura & Nei, 1993). The trees with the highest log likelihoods are shown. The percentage of trees in which the associated taxa clustered together is shown next to each branch. The trees are drawn to scale, with branch lengths measured in the number of substitu-tions per site. The trees were rooted using sequences from *C. butyricum* and *C. cylindrosporum*, members of *Clostridium sensu stricto* (cluster I). Evolutionary analyses were conducted in MEGA7 (Kumar *et al.*, 2016).
